# In Situ Eradication of Mature Oral Biofilm on Titanium Implant Surfaces Using Cold Atmospheric Plasma

**DOI:** 10.3390/dj13050210

**Published:** 2025-05-14

**Authors:** Markus Jörg Altenburger, Michael Eckhard Bergmann, Loic Alain Ledernez, Georgios Romanos

**Affiliations:** 1 Department of Orthodontics, Center for Dental Medicine, Medical Center—University of Freiburg, Faculty of Medicine, Albert-Ludwigs-University of Freiburg, Hugstetter Straße 55, 79106 Freiburg, Germany; 2 Department of Operative Dentistry and Periodontology, Center for Dental Medicine, Medical Center—University of Freiburg, Faculty of Medicine, Albert-Ludwigs-University of Freiburg, Hugstetter Straße 55, 79106 Freiburg, Germany; 3 Laboratory for Biomedical Microtechnology, Department of Microsystems Engineering (IMTEK), University of Freiburg, Georges-Koehler-Allee 103, 79110 Freiburg, Germany; 4 Freiburger Medizintechnik, Georges-Koehler-Allee 103, 79110 Freiburg, Germany; bergmann@frmed.de (M.E.B.); ledernez@frmed.de (L.A.L.); 5 Department of Periodontics and Endodontics, School of Dental Medicine, Stony Brook University, 106 Rockland Hall, Stony Brook, NY 11794, USA; georgios.romanos@stonybrookmedicine.edu

**Keywords:** cold atmospheric plasma, dental implant, disinfection, mucositis, peri-implantitis

## Abstract

**Objective:** This study evaluated the effectiveness of a new cold atmospheric plasma device (AmbiJet) for eradicating mature oral biofilm on titanium implant surfaces, aiming to improve decontamination methods for the treatment of peri-implant infections. **Material and methods:** Mature oral biofilms were grown on titanium disks placed in participants’ mouths. These disks were divided into control and plasma treatment groups. The AmbiJet device delivered plasma directly to the implant surface for 3 min per 20 mm^2^, utilizing the applicator nozzle and implant as electrodes. Biofilm reduction was quantified by counting colony-forming units (CFUs). **Results:** Cold plasma treatment rendered approximately 90% of samples bacteria-free. A > 6-log_10_ reduction (≈99.9999%) in bacterial load was achieved in 30% of samples, with an overall average reduction of 4.9-log_10_ across all treated samples. The temperature during treatment remained below 40 °C. **Conclusions:** Within the study’s limitations, cold atmospheric plasma effectively eradicates mature oral biofilm on titanium surfaces. This high disinfection efficacy is likely due to the combined action of reactive species and electrical phenomena, which does not cause significant temperature increases.

## 1. Introduction

Peri-implantitis is an inflammatory oral disease with increasing prevalence and incidence in industrialized nations. At the patient level, the onset of peri-implantitis (bone loss > 0.5 mm) was reported to increase from 70% (after two years) to 81% (after three years) [1]. In a study with a Swedish population, 59% of implants presented moderate/severe peri-implantitis (bone loss > 2 mm) after 9 years [2]. Several studies showed 100% implant-related mucositis in patients [3,4]. One cause for the inflammation of the surrounding tissues is the bacterial colonization of the implant and abutment surfaces. This inflammatory change in the tissue surrounding the implant allows the bacteria to spread further on the implant and attack the bone around the implant. This process continues until the bone is destroyed to such an extent that it can no longer provide the necessary supporting function for the implant. The implant, including the connected crowns, bridges and prostheses, must be removed. This results in renewed toothlessness in this area. The number of required peri-implantitis treatments is estimated to grow at a rate of about 13% every year [5].

Current attempts to stop the inflammatory response aim at cleaning the implant surface and eliminating the biofilm. Additionally, practitioners may attempt to regrow the bone, which also requires the implant surface to be free of bacteria. The etiology of periimplantitis is multifactorial, but the path to its management involves the removal of bacteria, which is insufficient with current methods [6]. Furthermore, mechanical damage occurs at the implant surface when using metallic curettes, ultrasonic devices, or even plastic-covered devices and air–powder instrumentation [7,8,9]. It also releases titanium particles from the implant, leading to renewed inflammation around the implant [10,11,12,13]. Therefore, chemical and/or pharmacological agents are used. However, the adjuvant administration of systemically or locally administered antibiotics only has a limited effect on biofilms [14,15]. In addition, side effects such as cytotoxicity [16,17], the extrusion of chemicals in healthy tissues [18,19], intolerance, or bacterial resistance to the antibiotic can occur, with many detrimental consequences [20,21,22,23,24,25,26].

Plasma is a multicomponent system that comprises particles that can contribute to the effective decontamination of surfaces by deactivating, killing, or disintegrating microorganisms such as bacteria and viruses [27,28]. Several devices with varying designs and technical realizations have been tested, aiming for a plasma application in dentistry. Gherardi et al. and Wu et al. reviewed the progress of cold plasma applications for dentistry [29,30]. Cold atmospheric-pressure plasma (CAP) can be used at room temperature and was shown to be very effective in increasing hydrophilicity [31], wound healing [32], and cell migration [33], even promoting osseointegration [31,34,35,36,37]. Furthermore, processes such as angiogenesis, microcirculation, changes in the pH value in the tissues, and further positive effects are boosted, thus promoting the healing process [38]. However, most are not efficient enough, showing only a partial removal of the biofilm [39,40,41,42]. Furthermore, these studies were performed with laboratory research devices that cannot be used in dental practices. Translating such a laboratory device into a medical product requires two major features: (1) the device must have a design that enables easy and effective (i.e., fast) use by dentists in patients’ mouths (i.e., small and handy); (2) the device must comply with a large number of regulatory requirements (ISO, DIN, IEC standards, etc., as well as the Medical Device Regulation (EU) 2017/745 in Europe). AmbiJet is a device based on electrical plasma that has been designed specifically for dentistry and fulfills legal requirements. Preliminary results showed that it effectively kills 10^6^-seeded aerobic and anaerobic bacteria after an application time of 1 min per 10 mm^2^; it does not lead to bacterial resistance and the target material remains under 40 °C [43].

By providing a controlled yet realistic environment, in situ studies offer a practical and efficient alternative to in vivo methods, making them a valuable tool in clinical research. Allowing the control of experimental variables, in situ studies can be conducted with a smaller group of participants over a shorter duration. This methodology is particularly advantageous for trials involving products that are not yet CE-certified, as it minimizes risks to patients. The aim of this work was therefore to test it in terms of disinfection efficacy in an in situ setup.

## 2. Materials and Methods

### 2.1. Study Design

**Figure 1:**
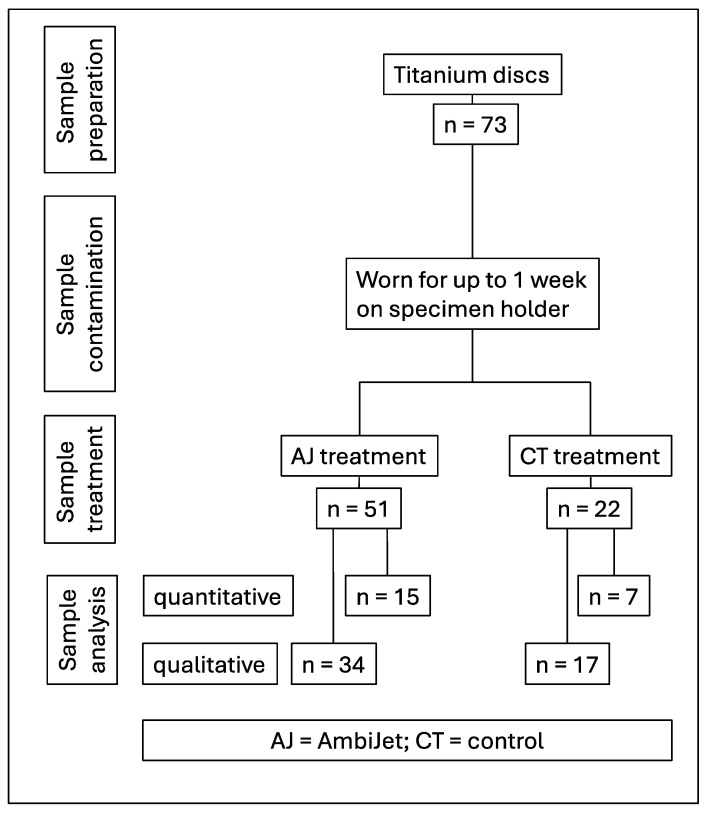
The design of the study with the sample size for each experiment.

**Figure 2:**
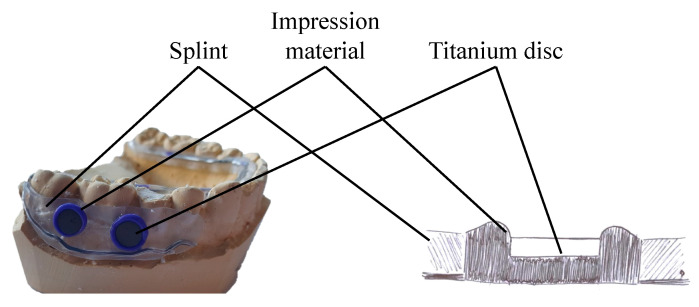
Picture (**left**) of a specimen holder with titanium disks; schematic drawing (**right**) in a cross-section. The impression material forms a ring around the disk to promote biofilm growth.

The design of the study with the sample size for each experiment is displayed in Figure 1. The study was part of a sequence of studies. The sample size was estimated by interpreting the results of preliminary in vitro studies. In total, 73 disks were used. Qualitative tests were performed using 15 plasma samples and 7 control samples. Quantitative tests were performed using a further 34 plasma samples and 17 control samples. Participants wore braces (individual fabrication, dental laboratory) with up to 6 titanium disks for several days, simulating the natural contamination of dental implants by native biofilm (Figure 2). The specimens were removed from the brace by a dentist and randomly stored in Eppendorf cups for processing by a technical assistant: in arm 1, the samples were rinsed only with water and formed the control group; in arm 2, the samples were rinsed with water and treated with plasma. The hypothesis stated that the plasma treatment of the biofilm-covered disks leads to a mean reduction in the bacteria by at least 99%. The null hypothesis was presented that the plasma treatment had no effect on the bacterial load of biofilm-covered titanium disks.

**Figure 3:**
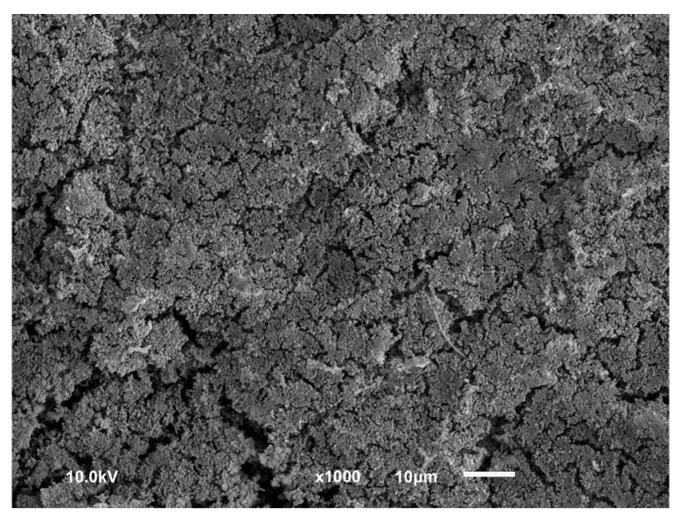
Scanning electron microscope picture of biofilm-covered titanium surface.

Impressions of the lower jaw were taken; plaster cast models and specimen holders were fabricated (Orthocryl, wire ø 0.9 mm spring hard, both: Dentaurum, Ispringen, Germany). Titanium disks (ø 5 mm, sandblasted and etched), fabricated and provided by an implant manufacturer (name undisclosed at its request), were inserted into oral splints (Panasil Initial Contact X-Light, Kettenbach, Huntington Beach, CA, USA) (Figure 2). Each splint could hold up to 3 samples on each side. The splints were worn by the participants for at least 21 h a day over a period of up to one week to allow biofilm growth (Figure 3). The splints were removed at mealtimes and for oral hygiene. During the times the splints were outside the mouth, they were kept in moist conditions. Normal oral hygiene was possible with a toothbrush, fluoride-containing toothpaste, and interdental plaque control, such as dental floss or interdental brushes. Antibacterial mouth rinses were not permitted.

### 2.2. Subject Selection

Prior to the study, all documents were positively reviewed by the ethics committee of the Albert-Ludwigs Universität Freiburg (Application no. EK-Freiburg: 21-1660; approved on 22 February 2022). The subjects were recruited by advertisements at the university. All subjects gave written consent for participation and fulfilled the inclusion—without violating the exclusion criteria. All subjects agreed to be compliant with the study protocol. All subjects underwent dental and periodontal inspection.

For the selection of the participants, the following inclusion criteria had to be fulfilled:

- Subjects must have an age between 18 and 60 years.
- No general illnesses relevant to the study.
- Subjects must have at least 20 teeth and acceptable oral hygiene (brushing at least twice a day).
- Able and willing to give written consent to participate in the study.
- Healthy or conservatively, prosthetically, and periodontally restored dentition.
- Willingness not to use antibacterial oral hygiene products (stannous fluoride, CHX, triclosan) during the study period.
- The salivary flow rate of the stimulated saliva should be at least 0.7 mL/min (determined by collecting saliva for one minute while chewing on a piece of paraffin).

The following exclusion criteria must not be violated:

- Dental treatments or other medical treatments in the oral cavity during the study period.
- Known allergies to previously used oral hygiene products and/or therapeutic products and/or dental materials used in the mouth or throat.
- Non-physiological tooth mobility.
- Pathological changes in the oral mucosa or gingiva.
- Excessive plaque formation.
- Eating disorders such as bulimia or anorexia nervosa.
- Use of antibiotics or chemical plaque treatments such as mouth rinses or varnishes
- Pregnancy or breastfeeding.
- Consumption of alcoholic beverages with more than 10% alcohol by volume (in their pure form or as a mixed drink).
- Past or current risky or pathological alcohol consumption—less than 2 alcohol-free days per week and/or more than one standard glass for women or 2 standard glasses for men of alcoholic beverages per day—as recommended by the government BZgA (Bundeszentrale für gesundheitliche Aufklärung).

### 2.3. Plasma Device

**Figure 4:**
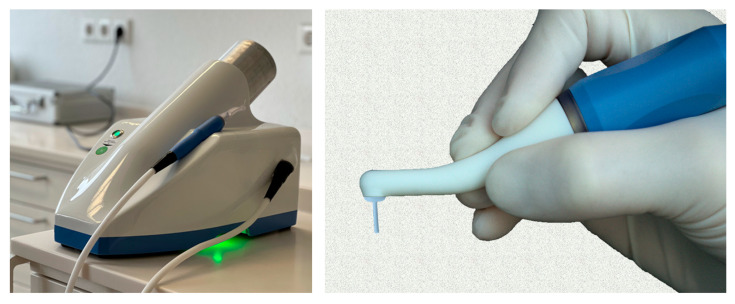
Photograph of cold plasma device (AmbiJet) and applicator based on ergonomics of dental instrument.

AmbiJet (Freiburger Medizintechnik GmbH, Freiburg i. Br., Germany) consists of a base unit with an integrated gas cartridge (helium), a handpiece, and applicators. The design of the handpiece with the applicator is based on the ergonomics of standard dental instruments (Figure 4). The target (disk or dental implant) is contacted electrically and builds the counter-electrode. Thus, the generated pulsed plasma is guided to the target by the electric field without becoming in contact with the surrounding soft and hard tissues. The plasma ignites only when the nozzle of the applicator is close enough to the sample. The plasma remains at a temperature below 40 °C.

### 2.4. Treatment

After the wearing period of splints, the samples were removed from the splints using sterile tweezers, briefly rinsed with water, not mechanically cleansed, and then divided into control group and plasma group. There was a break of at least 3 days between wearing periods during which no splint was worn. This was to avoid excessive strain on the participants and to prevent possible mechanical irritation of the oral mucosa caused by wearing the sample holders for long periods. At the end of the clinical phase of the study or if participation in the study ended prematurely, a final oral examination took place. At each visit, when the splints were inserted, at follow-up appointments, and when the splints were removed, the subject’s state of health, changes in medication intake, and any adverse events that may have occurred were documented.

**Figure 5:**
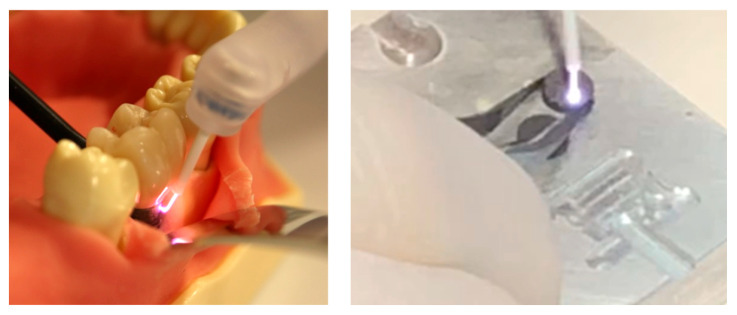
Picture (**left**) of plasma showing how it is guided to the dental implant thanks to the electrical field. Picture (**Right**) of plasma ignited against a titanium disk.

The samples from the control group were left untreated. Disks of the plasma group were placed on a metal carrier to ensure contact with the counter electrode (see Figure 5). The area exposed to the oral cavity was treated with the plasma jet. The surface area was 20 mm^2^; the treatment lasted 3 min.

Note that the nozzle of the applicator does not need to be perpendicular to the surface: by design, the plasma is guided to the surface of the implant as shown in Figure 5 (Left). In a real treatment, the dentist would clean the region around the implant (incl. pus) before applying a plasma treatment. Optionally, the dentist would perform an open-flap intervention to access the implant. 

### 2.5. Microbial Analysis

For the microbial analysis, the specimens were placed in cell culture plates. A microbial analysis was performed to evaluate the effectiveness of the cold plasma device. A distinction was made between a qualitative and quantitative analysis.

For the qualitative analysis, samples were taken from treated titanium disks (plasma group) and from untreated titanium disks (control group). These samples were then stored in culture medium for up to 72 h to detect contamination by bacterial turbidity of the samples. A yes/no decision was then made and documented.

In the quantitative analysis, the reduction in the bacterial contamination was quantitatively determined by colony forming unit (CFU) counting, as summarized in the following points:

- After treatment, wettened paper points (using tryptic soy broth, TSB) were used to wipe the treated or reference surfaces to collect bacteria off the surfaces.
- The paper points were transferred to Eppendorf tubes containing TSB and vortexed for 10 s.
- A dilution series was prepared for the CFU counting.
- A defined volume of the respective solutions was applied on agar plates and spatulated on Müller–Hinton agar in Petri dishes.
- The Petri dishes were incubated at 37 °C for two days to allow the bacteria to form colonies.
- The CFUs were then counted. The more of these CFUs were counted, the more bacteria were present in the detection solution and consequently in the samples, which indicates that there was a higher bacterial load on the samples.

### 2.6. Mathematical Analysis

The results were documented, and mean values were calculated over the dilution series and the results compared with the values of the control group. The mathematical analysis was performed using a commercially available software program (PASW Statistics 19.0, SPSS Inc., Chicago, IL, USA). Mean values and standard deviations were calculated for each variable and group using the patient as the statistical unit.

## 3. Results

### 3.1. Qualitative Results

In the qualitative analysis, the samples taken were tested for sterility. This means that a single germ was enough to make the result appear positive (contaminated). This is an enormous challenge for the device, but also for the methodology of the analysis. In everyday clinical practice, absolute sterility is unlikely due to the presence of saliva or breath. Results shown in Table 1 therefore impressively demonstrate the effectiveness of the device. No germ could be detected on 14 out of the 15 treated samples after 1 min of treatment.

The reference samples confirm that the samples were contaminated before the plasma treatment. The different time stamps (1 to 3 days) show that even slow-growing germs are not detectable, and that the bacterial reduction was absolute in 14 out of 15 samples.

### 3.2. Quantitative Results

**Figure 6:**
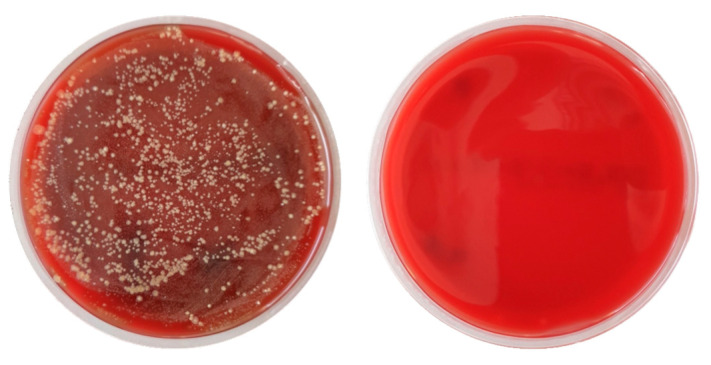
Photograph of two Petri dishes: plating out of samples taken from untreated titanium disks (**left**) and treated titanium disks (**right**). For better visualization of CFUs, Columbia blood agar was used in this case.

After the incubation period, the disk contamination was evaluated. Figure 6 shows the effect of the treatment in an impressive way. Untreated: the number of CFUs can only be counted and calculated via a dilution series. Plasma-treated: no CFUs could be detected for most samples.

**Figure 7:**
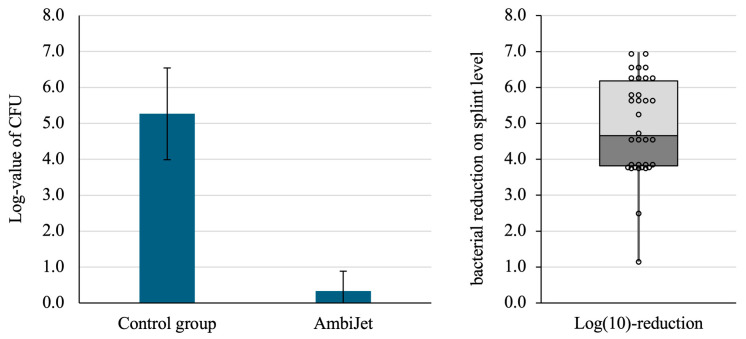
Averaged (**left**) bacterial reduction between non-treated samples (*n* = 17) and treated samples (*n* = 34) (*p* < 0.05). Boxplot (**right**) and superimposed scatter plot of the bacterial reduction.

Figure 7 (Left) displays the averaged CFU counting for all control disks and for all treated disks. On average, the reduction in CFU count is 4.9-log(10). Obviously, depending on the bacterial load on the disks of the control group, complete eradication (sterile surface) can be reached by bacterial reduction of under 6-log(10). The percentage of samples for which no CFUs could be detected was actually 88.2% (30 out of 34), which corresponds very well with the results of the qualitative analysis.

Figure 7 (right) shows the distribution of bacterial reduction on splint-level, i.e., the CFU counting of plasma treated disks are compared to their respective control disks from the same splint. A scatterplot is superimposed to the boxplot to help visualize the plasma effect. The following elements can be deduced:

- The first quartile is long because 2 disks out of 34 delivered lower values.
- Out of 34 treated disks, 10 samples (ca. 30%) displayed a bacterial reduction ranging from 6-log(10) to 7-log(10). This is only obtained with control disk with a very high initial contamination.
- The samples displaying a ca. 4-log(10) reduction are bacteria-free. The bacterial reduction is lower because the initial contamination was lower for those splints.

## 4. Discussion

The aim of this study was to evaluate the effectiveness of a cold plasma device in reducing the bacterial contamination on titanium disks. The applied study design has already proven to be suitable to cultivate the oral biofilm in situ and to investigate influences on the oral microbiota in previous studies [44,45,46]. Thanks to the in situ setup, the synergistic effect of the different bacteria organized in a mature oral biofilm as well as the possible effect of the inter-individual composition of the biofilm are taken into account. Therefore, the present in vivo study protocol reflects the clinical situation better than in vitro setups with mono- or multispecies biofilms. Moreover, thanks to the better control of variables, the same quality of results can be achieved with a much smaller group of participants in a much shorter period of time than with RCT studies. The in situ study also produces data without putting the study subjects at risk.

In the present study, in about 90% of cases, zero bacteria were found after the plasma treatment. This was observed for the sets of experiments analyzed using the two different methods. Even with an initial bacterial burden of >6-log(10), the disks were free of bacteria after the treatment. In fact, reductions in bacterial contamination of up to >6-log(10) levels were achieved in 30% of samples. The average bacterial reduction of 4.9-log(10) is actually limited by the initial bacterial burden.

This demonstrates the high effectiveness of the device and distinguishes it from other treatment options currently available or recommended in the literature. The electrical plasma has a physical mode of action and is therefore effective for all bacteria, even on a grown mixed biofilm. In contrast, brushes, water pressure, and powder water jet devices only work by treating the surface mechanically, which does not lead to a sufficient reduction in the bacterial load. Chemical agents such as disinfectants or antibiotics can be effective against individual bacterial strains but have a very limited bactericidal effect on biofilms.

Currently, there are no data available that compare electrical plasma disinfection with other available methods in dentistry. Mechanical debridement with curettes and brushes or powder water sprays do not lead to disinfection. Thus, the biofilm bacteria residing in the crypts of the roughened titanium surface will not be removed or inactivated. Emerging biomechanical technologies using galvanic elements or bactericidal gels may be effective alternative methods. Future investigations should therefore focus on comparing those new technologies in terms of efficacy and safety.

## 5. Conclusions

The disinfecting effect of cold atmospheric plasma has been reported in the literature when using experimental devices in laboratory setups. However, those devices have not been transferred to products that comply with standards or meet regulatory requirements. Thus, previous results remain within the scientific realm. The cold atmospheric plasma device tested here fulfills mandatory regulatory requirements as well as ergonomic specification for easy intra-oral application. This study firstly shows that electrical plasma ignited by a medical device complying with medical standards is able to eradicate natural oral biofilm from titanium implant surfaces so that it may be used to treat periimplantitis effectively. To the authors’ knowledge, the efficacy demonstrated cannot be achieved with any other method currently available or used in dentistry. Limitations: The in situ study aims at evaluating the clinical performance of a device or method, and does not deliver insight on clinical safety. Therefore, further experimental work should include the study of the influence of plasma on human cells. Furthermore, a clinical study on a large scale of patients should be performed in the near future to assess the long-term efficacy and the clinical safety of the method.

## Figures and Tables

**Table 1:** Results of the qualitative analysis. The numbers are the number of samples.

		After 24 h	After 48 h	After 72 h
Plasma group	Negative (no bacteria)	14	14	14
	Positive (contamination)	1	1	1
Control group	Negative (no bacteria)	0	0	0
	Positive (contamination)	7	7	7

## Data Availability

All data presented in the paper is not openly available due to reasons of sensitivity. All data is available from the author upon reasonable request. All data is located in a controlled access data storage at the University of Freiburg.
